# Bridging the Gap Between Social Determinants and Health Profile: A New Stratification Tool for the Italian National Health Service

**DOI:** 10.3390/healthcare14111456

**Published:** 2026-05-25

**Authors:** Elvira Massaro, Irene Schenone, Daniela Amicizia, Francesca Marchini, Matteo Astengo, Federico Grammatico, Andrea Fiorano, Alexander Domnich, Donatella Panatto, Giancarlo Icardi, Filippo Ansaldi

**Affiliations:** 1 Department of Health Sciences, University of Genoa, 16132 Genoa, Italy; daniela.amicizia@unige.it (D.A.); panatto@unige.it (D.P.); icardi@unige.it (G.I.); filippo.ansaldi@unige.it (F.A.); 2 IRCCS Azienda Ospedaliera Metropolitana (AOM), San Martino Policlinic Hospital, 16132 Genoa, Italy; irene.schenone@aomliguria.it (I.S.); francesca.marchini@aomliguria.it (F.M.); matteo.astengo@aomliguria.it (M.A.); federico.grammatico@aomliguria.it (F.G.); andrea.fiorano@aomliguria.it (A.F.); alexander.domnich@aomliguria.it (A.D.)

**Keywords:** population health management, socioeconomic determinants of health, health equity, regional health planning

## Abstract

**Background/Objectives**: In Italy, the ongoing reform of primary healthcare (Ministerial Decree 77/2022) requires Health Districts to shift towards proactive, need-based resource allocation. Despite evidence of their role in shaping citizens’ health, socioeconomic deprivation indices remain rarely integrated into territorial planning frameworks. This study develops and validates a population-weighted analytical model linking area-level socioeconomic deprivation, territorial accessibility, and all-cause mortality across the entire Italian territory, with the aim of supporting evidence-based planning. **Methods**: All 7899 Italian municipalities were aggregated into 1175 territorial units defined by Health District boundaries and SNAI (National Strategy for Inner Areas) classification. A population-weighted multivariable OLS regression model was used to examine the association between socioeconomic indicators (educational deprivation, employment, household isolation) and the Standardized Mortality Ratio (SMR) for 2023–2024. **Results**: The model explained 72.5% of the variance in SMR across territorial units (adjusted R^2^ = 0.719; F = 116.5; *p* < 0.0001). Region of residence emerged as the dominant predictor. Educational deprivation showed the strongest positive association with mortality. While employment-related deprivation was inversely associated with SMR, household isolation showed a positive independent association with mortality. Residual mapping identified spatial clusters of excess mortality unexplained by socioeconomic factors, pointing to unmeasured determinants including environmental exposures and healthcare quality differentials **Conclusions**: Our model provides a replicable, evidence-based framework for identifying territorial vulnerability and prioritising healthcare resources at the Health District level. By benchmarking observed mortality against socioeconomic predictions, it enables planners to distinguish structurally driven excess mortality from potentially amenable mortality, supporting proactive, equity-oriented planning consistent with the objectives of Ministerial Decree 77/2022.

## 1. Introduction

The sustainability of modern healthcare systems is increasingly challenged by a profound demographic transition toward an aging population and a rising prevalence of multimorbidity [1]. To maintain equitable access and manage escalating costs, universal healthcare systems need to shift from reactive, hospital-centered care to proactive population health management (PHM). This approach involves stratifying populations according to specific health needs, enabling policymakers to optimize resource allocation and prevent morbidity and avoidable mortality [2].

Despite robust evidence that socioeconomic inequalities drive adverse health outcomes [3,4], traditional risk stratification models remain predominantly focused on clinical diagnoses [5]. While current comorbidity scores are reliable predictors of individual mortality, they frequently overlook the complex environmental and social drivers that dictate healthcare demand and service utilization at the community level [6]. Consequently, stratification based solely on clinical data risks producing an incomplete profile of a population’s actual burden of care, and fails to integrate socioeconomic deprivation and geographic accessibility within a unified framework [2,7].

In Italy, the recent reform of primary healthcare (DM 77/2022) aims to standardize care through the development of proximity networks, community-based facilities, and telemedicine services [8]. However, the effective implementation of these standards requires a fine-grained, multidimensional understanding of local health determinants, since a “one-size-fits-all” application of national standards may fail to address the specific vulnerabilities of heterogeneous territories [9].

Deprivation indices (DI) based on socioeconomic indicators such as education, employment, and housing have become essential tools for assessing territorial inequalities [10]. However, a key gap remains in translating these indices into actionable care pathways that also account for healthcare accessibility and geographic barriers [2].

Within the ongoing reorganization of territorial healthcare services in Italy, the National Strategy for Inland Areas (SNAI) plays a key role by classifying municipalities according to their distance from essential service hubs, including healthcare, education, and transport. This classification distinguishes central areas from progressively more remote territories, which face increasing physical and organizational barriers to access. The National Strategic Plan for Inland Areas (PSNAI) 2021–2027 identifies healthcare accessibility as a pillar of territorial cohesion, particularly in peripheral areas [11]. In this context, the SNAI classification could be interpreted as a policy-relevant proxy for geographical and organizational deprivation, capturing structural constraints not reflected by socioeconomic indicators alone and complementing traditional deprivation indices to support more nuanced and place-based health planning.

Building upon existing literature [12,13,14] and established Italian deprivation frameworks [15], this study adopts a population-weighted area-level analytical approach using publicly available data from all Italian regions to examine the association between socioeconomic deprivation, territorial accessibility and mortality, contributing with a new tool for regional and local planning.

## 2. Materials and Methods

### 2.1. Study Design and Setting

We carried out an ecological study based on multi-source data linkage of publicly available data, with aggregated territorial areas as the unit of analysis. Reporting followed the STROBE (Strengthening the Reporting of Observational Studies in Epidemiology) checklist (Table S1). The analysis encompassed the entire Italian population (58,971,031 residents) based on 2024 records from the Italian National Institute of Statistics (ISTAT). For the purposes of territorial planning and consistent with the ecological area-level design of the study, municipalities were aggregated within territorial units. This approach was designed to enable a multidimensional assessment of territorial vulnerability, even recognizing the inferential limits of aggregated, area-based analyses, aiming to reflect the operational scale of local healthcare organization since, in Italy, Health Districts are the primary sub-regional administrative units responsible for primary healthcare delivery, as defined by D.Lgs. 502/92 (each District typically encompasses multiple municipalities). Municipalities were clustered on the basis of Health District boundaries and were subsequently characterized according to SNAI classification, which captures gradients in territorial accessibility to services (A: Pole; B: Intermunicipal Pole; C: Belt; D: Intermediate; EF: Peripheral/Ultra-peripheral) [11]; they were thus aggregated into “policy-relevant small areas” to preserve their data while ensuring statistical robustness, by accounting for local contextual factors and the specific administrative jurisdictions responsible for public health interventions. The 1175 territorial units were defined by aggregating contiguous municipalities sharing the same SNAI classification within a given Health District boundary: in 123 cases, the territorial unit coincided perfectly with the institutional Health District (i.e., all municipalities of the District shared the same SNAI class). In cases where a District contained municipalities with different SNAI classifications, it was split into multiple territorial units.

In contrast to previous research that opted to exclude small municipalities due to potential data volatility [12,13], our approach intentionally included them, since small and often isolated communities provide critical insights into territorial vulnerability.

### 2.2. Data Sources and Variable Definition

The study population included all residents in Italy, based on ISTAT 2024 records. All territorial units had complete information on socioeconomic indicators and mortality data were considered eligible for inclusion. Socioeconomic data were derived from the 2021 Permanent Census of Population and Housing, the most recent data available, and were initially calculated at the census section level: municipalities were defined by aggregating their corresponding census sections [16]. Following this, a further aggregation was performed to align the data with territorial units defined by institutional Health District boundaries, cross-referenced with the National Strategy for Inner Areas (SNAI) classification [17]. Mortality data for 2023 and 2024 were obtained from ISTAT [18]. The socioeconomic profile of each territorial unit was defined using modified six area-level socioeconomic indicators selected from established Italian deprivation frameworks, including the deprivation index originally developed by Caranci et al. [19]. Six key indicators were calculated for each unit:

- L1 (Education): % of population ≥9 years with education below upper secondary school;
- L2 (Employment): % of the working-age population (15–64 years) that is employed, calculated as the share of employed residents within the total population of the same age group;
- L3 (Citizenship): % of foreign residents;
- L4 (Household density): average number of occupants per housing unit;
- L4a (Isolation): % of housing units with a single occupant;
- L4b (Overcrowding): % of housing units with 5+ occupants.

Building upon this foundation and drawing inspiration from recent European multidimensional frameworks [20,21], this study refined the selection of variables to capture a broader spectrum of social inequality. The study analytical workflow is reported in Table S2.

### 2.3. Outcome

The primary outcome was the Standardized Mortality Ratio (SMR). Annual SMRs were calculated separately for 2023 and 2024 as the ratio of observed to expected deaths (standardized by age), with national age-specific mortality rates serving as the reference. To minimize annual fluctuations, the final indicator was derived as the average of the two annual SMRs. To assess mortality differences across levels of deprivation, SMRs were grouped into quintiles.

### 2.4. Statistical Analysis

Data cleaning and management were initially conducted using Microsoft Excel (version 1808; Microsoft, Redmond, WA, USA) following predefined and standardized procedures. The dataset was screened for duplicate records, inconsistencies and out-of-range values. Duplicates were identified using unique subject identifiers and removed prior to analysis. Logical consistency checks were performed to identify implausible or contradictory entries, which were verified against source data when available. Categorical variables were standardized, and continuous variables were assessed for distributional properties and extreme values. All procedures were documented to ensure transparency and reproducibility.

Statistical analyses were performed using JMP Pro 17 (SAS Institute Inc., Cary, NC, USA). Descriptive statistics were computed for all variables, with quantitative variables summarized using mean, median and standard deviation (SD) and categorical variables reported as absolute and relative frequencies (percentages).

Bivariate associations between socioeconomic indicators (L1–L4b) and the SMR for the period 2023–2024 were assessed using Pearson correlation coefficients.

To examine the association between socioeconomic deprivation indicators and the mean SMR, population-weighted ordinary least squares (OLS) regression model was fitted, using SMR as the dependent variable and socioeconomic indicators as independent variables. To account for differences in the precision of area-level mortality estimates across spatial units with substantially varying sizes, models were fitted using analytic weights proportional to the resident population of each territorial unit. Weights were defined as the resident population of each territorial unit. Specifically, in the area-level models, weighted least squares regression was applied using the resident population of each territorial unit as an analytic weight, ensuring that more populous areas contributed proportionally more to parameter estimation. This approach accounts for differences in the precision of area-level mortality estimates across units with substantially varying population sizes. Weighting was necessary to avoid assigning the same influence to small territorial units (e.g., 1000 residents) as to large metropolitan areas (e.g., 2 million residents), thereby enhancing the robustness and policy relevance of the model’s estimates. Regression coefficients were reported on the original scale of the deprivation measures and represent the estimated change in SMR associated with a one-percentage-point increase in each indicator. Model parameters were estimated using ordinary least squares within the “Fit Model” platform and diagnostic plots were used to assess model assumptions.

Distributional assumptions of the OLS model, including linearity, independence of errors, homoscedasticity and normality of residuals, were evaluated using graphical diagnostics. Multicollinearity was assessed using the Variance Inflation Factor (VIF) and pairwise correlation coefficients. Variables with VIF > 10 or |r| > 0.7 were excluded from the final model. Model performance was evaluated using the coefficient of determination (R^2^), with statistical significance set at *p* < 0.05. As the lack of calibration may lead predicted SMR values to deviate from the national mean of 1.00, increasing sensitivity to stochastic noise in small areas and limiting suitability for direct use in resource allocation, these limitations could be addressed by adopting the following calibrated fixed-effects (FE) framework:

SMR_{predicted, i} = β_0_ + β_1_(L1_i) + β_2_(L2_i) + β_3_(L4a_i) + γ_{SNAI, k} + δ_{Region, j}

In order to assess the robustness of the main findings, sensitivity analysis were performed. SNAI area classification was retained in the main area-level model; however, to address potential concerns regarding its overlap with the territorial classification strategy, the model was re-estimated after excluding SNAI area classification from the adjustment set.

## 3. Results

As of January 2024, the Italian territory comprised 7899 municipalities, characterized by significant heterogeneity in population size and mortality outcomes. The average population per municipality was 7465 residents, though a median of 2392 revealed a distribution heavily skewed by small centers, with sizes ranging from a minimum of 33 to a maximum of 2,751,747 residents in the capital.

At baseline, mean educational deprivation (L1) was 52.43%, the employment rate (L2) was 66.85%, and single-occupant households (L4a) accounted for 37.48%.

Detailed descriptive statistics for the aggregated units are provided in Table 1 and Table S3.

**Figure 1:**
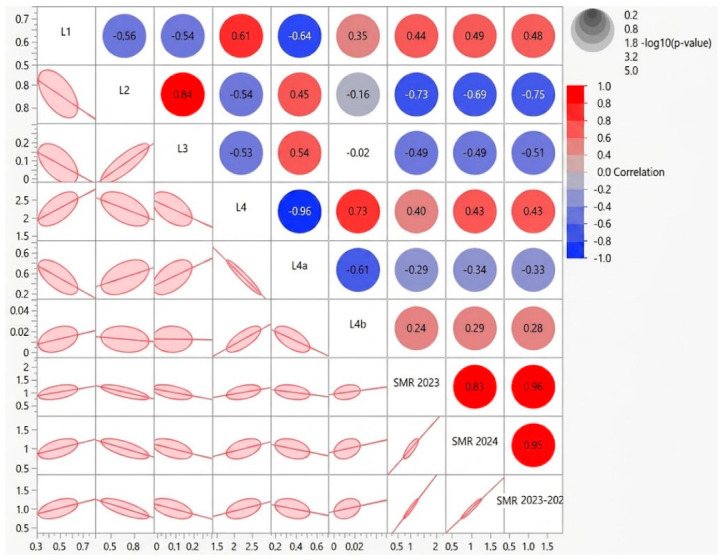
Population-weighted correlation matrix of socioeconomic indicators (L1–L4b) and Standardized Mortality Ratio (SMR) 2023–2024. Population-weighted correlation matrix of socioeconomic indicators (L1–L4b) and the Standardized Mortality Ratio (SMR) for 2023–2024. Coefficients (−1 to +1) reflect the strength and direction of associations, with population weighting giving greater influence to more populous areas. The matrix highlights relationships among indicators and their links to mortality, aiding interpretation of socioeconomic gradients and potential multicollinearity.

Population-weighted bivariate analysis identified distinct associations between socioeconomic indicators (L1–L4b) and the Standardized Mortality Ratio (SMR) for the 2023–2024 period (Figure 1 and Table S4).

Low educational (L1) showed a moderate positive correlation with SMR (r = 0.484), while employment rate (L2) and proportion of foreign residents (L3) were both negatively correlated with SMR (r = −0.746 and r = −0.51, respectively).

A high degree of collinearity was observed between L2 and L3 (r = 0.84), suggesting that these indicators capture a shared socioeconomic construct; to ensure model parsimony and prevent multicollinearity, L3 was excluded from multivariate analyses. Moreover, redundant covariates (L4 and L4b) were likewise excluded from further modeling. Although household isolation (L4a) demonstrated a negative bivariate association with SMR (r = −0.33), its coefficient was positive (β = +0.29) in the multivariate model. This pattern consistent with suppressor variable dynamics: in the bivariate setting, L4a partially captures the lower mortality of rural, less-crowded Northern areas; once educational deprivation, employment, and region are controlled, its independent contribution to excess mortality becomes apparent, suggesting a complex interaction between isolation and rural–urban mortality gradients.

The relationship between territorial peripherality, as defined by the PSNAI and mortality showed a pronounced national gradient between territorial peripherality and SMR. Overall, SMR increased progressively from 0.981 in urban “Poles” (Class A) to 1.053 in “Peripheral/Ultra-peripheral” areas (Class EF), suggesting higher mortality in more remote and less accessible territories.

**Figure 2:**
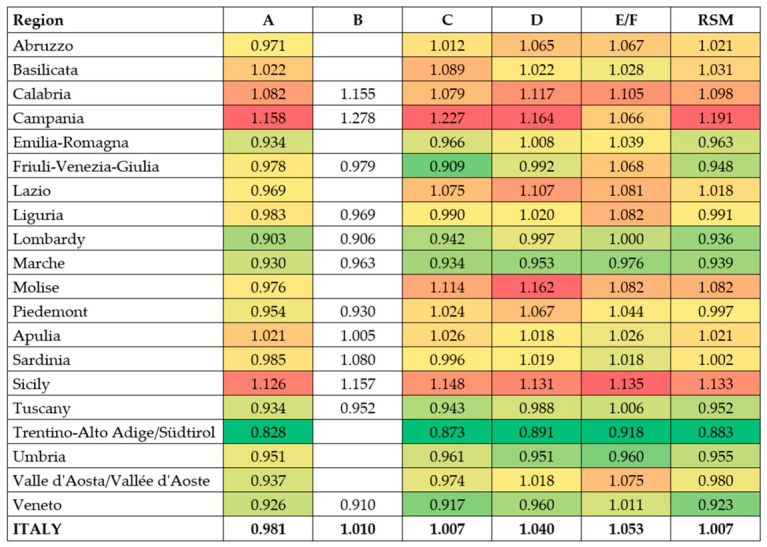
Mean Standardized Mortality Ratio (SMR) by SNAI (National Strategy for Inner Areas) classification and region in Italy. Territorial units are grouped according to SNAI categories which reflect varying levels of access to essential services and are aggregated at the regional level. The figure illustrates spatial variability in mortality, highlighting disparities between central and peripheral areas across regions. The figure uses a green-to-red color scale to represent the distribution of values from lowest to highest.

Stratification by region revealed significant geographical heterogeneity (Figure 2). Regions such as Lombardia, Emilia-Romagna, and Veneto exhibited a monotonic increase in mortality as one moved from urban centers to remote areas. In Lombardy, for instance, SMR values shifted from 0.903 in Class A to 1.000 in Class E/F, suggesting that the centralization of services created a protective “urban advantage”.

Conversely, Southern regions such as Campania and Sicily displayed markedly different patterns. In Campania, urban poles (Class A) showed very high SMR (1.158), exceeding even their most remote counterparts (Class E/F: 1.066). Similarly, Sicily presents a “flat” distribution with consistently elevated SMR across all territorial classes, indicating a systemic disadvantage that transcended geographical accessibility.

The population-weighted multivariable linear regression with fixed effects for region and SNAI area resulted in a substantial proportion of the variance in mortality across Italian territorial units (R^2^ = 0.725; adjusted R^2^ = 0.719; F = 116.5; *p* < 0.0001).

After adjustment for regional and SNAI fixed effects, all selected socioeconomic indicators were strongly associated with SMR.

Educational deprivation remained strongly and positively associated with higher SMR (L1: β = 0.59; 95% CI: 0.49 to 0.68; *p* < 0.0001), indicating that populations with lower levels of education tend to experience substantially higher mortality. This relationship might reflect multiple underlying mechanisms, including reduced health literacy, limited access to resources, and long-term socioeconomic disadvantage.

In contrast, employment-related deprivation showed an inverse association with SMR (L2: β = −0.38; 95% CI: −0.47 to −0.29; *p* < 0.0001). This counterintuitive finding may suggest that the indicator captures complex labor-market dynamics—such as the presence of economically active but vulnerable populations—or that it overlaps with other dimensions of deprivation in ways that require further investigation. Social isolation was also positively associated with mortality (L4a: β = 0.29; 95% CI: 0.20 to 0.38; *p* < 0.0001), reinforcing evidence that weaker social ties and reduced community support can negatively affect health outcomes, potentially through both psychosocial stress and reduced access to informal care. The global test for SNAI fixed effects was statistically significant (F = 4.33; *p* = 0.0018), suggesting that territorial classification contributed independently to explaining SMR variability after adjustment for socioeconomic indicators and region. Among SNAI categories, only category D showed a statistically significant positive coefficient (β = 0.01; 95% CI: 0.00 to 0.02; *p* = 0.0153) (Table 2).

Region emerged as a primary determinant (Log-worth = 95.44). A persistent North–South gradient was present in the adjusted estimates: as for Southern Regions significant positive estimates were observed for Campania (0.123), Sicily (0.032), and Calabria (0.025). Conversely, regions such as Sardinia (−0.090), Marche (−0.032), Tuscany (−0.021), and Lombardy (−0.014) exhibited protective effects relative to the national average (Tables S5–S7).

The sensitivity model showed only a minimal reduction in explanatory performance compared with the main model, with an R^2^ of 0.721 and an adjusted R^2^ of 0.716; the overall model remained highly significant (F = 135.38; *p* < 0.0001) (Tables S8–S10). Educational deprivation remained positively associated with SMR (L1: β = 0.55; 95% CI: 0.47 to 0.64; *p* < 0.0001), employment-related deprivation remained inversely associated with SMR (L2: β = −0.36; 95% CI: −0.44 to −0.27; *p* < 0.0001), and social isolation retained a positive association with mortality (L4a: β = 0.24; 95% CI: 0.16 to 0.31; *p* < 0.0001).

Overall, these findings indicate that the associations between deprivation indicators and SMR were robust to the exclusion of SNAI fixed effects.

Table 2 presents the results of population-weighted multivariable linear regression with fixed effects for region and SNAI area.

**Figure 3:**
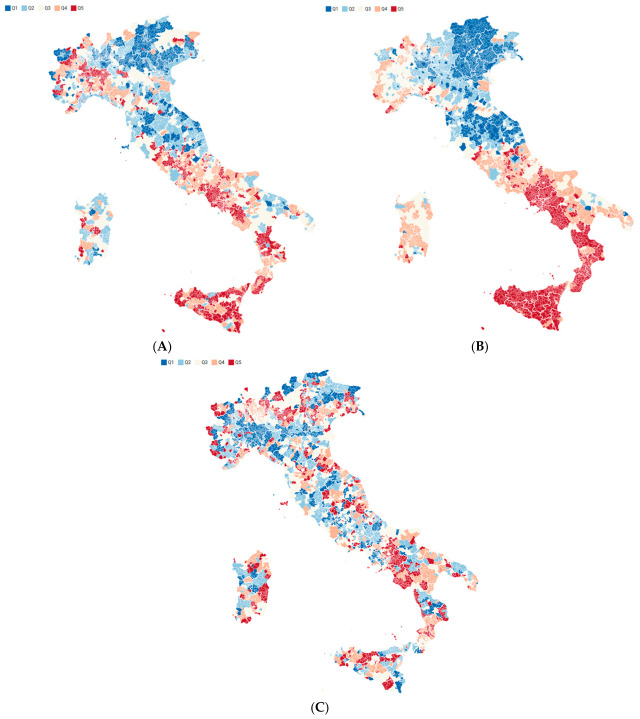
Geographic distribution of the observed Standardized Mortality Ratio (SMR) in panel A, the model-predicted SMR across Italian territorial units in panel B, and the residuals map in panel C. Quintile classification and respective values: (**A**) Q1: 0.48–0.93 (dark blue); Q2: 0.93–1.00 (light blue); Q3: 1.00–1.06 (beige); Q4: 1.06–1.11 (orange); Q5: 1.11–3.66 (red). (**B**) Q1: 0.82–0.96 (dark blue); Q2: 0.96–0.99 (light blue); Q3: 0.99–1.03 (beige); Q4: 1.03–1.09 (orange); Q5: 1.09–1.28 (red). (**C**) Q1: −0.85–−0.07 (dark blue); Q2: −0.07–−0.02 (light blue); Q3: −0.02–0.01 (beige); Q4: 0.01–0.05 (orange); Q5: 0.05–0.62 (red).

Finally, the comparison between the SMR of different territorial units and the predicted mortality based on socioeconomic drivers was performed, as shown in Figure 3. Maps display the spatial distribution of the observed SMR (A), the SMR predicted by the model for each territorial unit (B) and the residual map (C). Values are grouped into quintiles (Q1–Q5), where Q1 indicates the lowest and Q5 the highest SMR.

## 4. Discussion

1. The Italian National Health Service (NHS) is currently navigating a profound demographic transition characterized by an aging population and a rising prevalence of multimorbidity. While the DM 77/2022 regulatory framework aims to modernize the system through proximity networks, its success is hindered by resource allocation models; currently, approximately 60% of the National Health Fund relies on simple per-capita quotas, with only 40% being age-weighted, so the imbalance fails to align financial flows with the actual health needs of an increasingly fragile population [22]. Since decisions regarding resource allocation have the potential to reduce unwarranted geographic variation, and given that chronic diseases accumulate more rapidly in populations with lower socioeconomic status, by integrating socioeconomic and geographical variables, the model offers a framework for shifting from disease-centric to vulnerability-adjusted modeling, allowing the NHS to transition from reactive treatment to proactive population management [23,24,25]. As an illustrative example, the model could be incorporated as an equity adjustment to per-capita funding, whereby areas in the highest deprivation quintile (Q5) receive a positive corrective weighting to reflect greater structural health needs, while lower-deprivation areas remain closer to the baseline allocation. This should be interpreted as a conceptual policy application rather than a prescriptive funding rule.
2. Further evaluation and formal calibration would be required prior to any real-world implementation.
3. Our model explains 72.5% of the variance in mortality across 1175 territorial units, suggesting that socioeconomic and geographical variables are not merely contextual “noise” but primary drivers of health outcomes. The prominence of “region of residence” suggests that Italy’s decentralized healthcare structure significantly dictates mortality outcomes, with “excess risk” likely tied to systemic healthcare delivery challenges and other factors not analyzed and that likely reflects differences in healthcare organization, accessibility and quality across regions, as well as broader contextual factors such as the strength of social networks [26,27].

Importantly, while individual socioeconomic indicators may fluctuate at the micro-level (small municipalities), our aggregation provide a stable, significant dose–response gradient: as the multidimensional deprivation score increases, the excess mortality risk rises proportionally. This relationship is widely documented and supports the interpretation that cumulative disadvantage translates into poorer health outcomes [26,27].

Among the different dimensions, education emerged as the most potent driver of health inequality. Substantively, this suggests that educational disadvantage operates through multiple pathways, including lower health literacy, reduced ability to navigate healthcare systems, and prolonged exposure to adverse socioeconomic conditions. These findings are consistent with a large body of literature identifying education as a key determinant of health inequalities and premature mortality across different contexts [26,28]. From a policy perspective, this highlights the importance of targeted interventions aimed at improving health literacy and access to information, particularly within Community Houses and other primary care settings located in high-deprivation areas. Since, in our study, “employment” is inversely correlated with deprivation, the negative association observed for the employment-related dimension (L2) should be interpreted with caution. Rather than indicating a true protective effect, this result likely reflects how the indicator is constructed and possible residual confounding.

“Isolation” (L4a) demonstrated a significant association with increased mortality.

Other studies found similar findings where loneliness, social isolation and living alone were significant risk factors for mortality, particularly in older adults [29,30,31].

The mechanisms underlying this relationship likely include both psychosocial pathways (e.g., chronic stress, depression) and reduced access to informal care and support networks. From a policy standpoint, this underscores the need for interventions that strengthen social cohesion and community-based support, especially in areas with a high prevalence of vulnerable and aging populations.

Geographically, Northern regions like Lombardia and Veneto show a clear linear progression and mortality increases as one moves from urban “Poles” (Class A) to “Ultra-peripheral” areas (Class EF), denoting specific territorial disparities. In contrast, Campania and Sicily exhibit high SMR values even in metropolitan hubs, suggesting that Southern healthcare services suffer from a more systemic structural deprivation. The findings are consistent with a recent study showing that, as life expectancy trends in Italy changed after major reforms, slowing regional convergence and decentralization may have contributed to new regional differences in survival [32]. The absence of coordinated national programming represents a critical structural weakness of the Italian NHS that this model, among others, is trying to adress.

The comparison between observed and predicted SMR of different territorial units yelded important observations. In Southern regions such Campania and Sicily, the maps show high concordance, confirming that mortality in these areas is heavily determined by structural deprivation (low education and employment). Furthermore, the model’s ability to identify “mismatches” between predicted and observed mortality provides systematic basis for prioritising areas where public health interventions are more needed. In industrial areas of the North-West, the model consistently underestimates mortality as observed in Casale Monferrato (Alessandria Province, Piedmont); this “excess risk” despite high socioeconomic status (L1—Education and L2—Employment) suggests a potential role of unmeasured factors like environmental stressors (e.g., air quality, industrial pollutants) as critical, unmeasured variables.

More broadly, the residual map (Figure 3C) reveals distinct spatial clusters of underestimation (positive residuals: observed SMR > predicted SMR) concentrated in parts of Campania, Lazio, and some northern industrial districts. These clusters point to unmeasured determinants—environmental exposures, healthcare access differentials, or lifestyle risk factors—that the socioeconomic model does not capture. Conversely, areas with negative residuals (observed SMR < predicted SMR) may reflect locally effective public health interventions or unmeasured protective factors and warrant further investigation as potential models of good practice. Given that region emerged as the dominant predictor, the model’s practical utility may be optimised when applied at the regional level, using region-specific reference rates and the residual map to identify districts where mortality deviates meaningfully from the expected socioeconomic profile.

While previous studies, such as the Italian Atlas of Mortality Inequalities [33], have exhaustively documented the epidemiological link between socioeconomic position and mortality, our work advances this discourse by translating epidemiological evidence into an operational tool for public health programming. By benchmarking geographical mortality net of age and crucial socioeconomic aspects, we allow the interpretation of mortality variations as potential indicators of healthcare performance and broader social and contextual influences. Where environmental explanations are absent, excess mortality in a specific district may represent amenable mortality: an unfavorable outcome that local healthcare action could have prevented or mitigated.

The “double burden” of social deprivation and territorial peripherality underscores that higher operational costs in remote areas—often viewed as inefficiencies—should be reframed as evidence-based investments.

As a practical illustration, a Health District scoring in the highest deprivation quintile for education and isolation (Q5) could prioritize deployment of mobile health teams and Community Health Houses (Case della Comunità) in underserved municipalities, consistent with DM 77/2022 objectives. In parallel chronic-disease screening and prevention programs could be targeted towards populations characterized by lower health literacy. Conversely, districts showing excess residual mortality despite low deprivation scores should be flagged for investigation of environmental or healthcare-quality factors, as these represent a different but equally actionable public health priority [34].

By incorporating social determinants of health into planning decisions, resources can be allocated more effectively to populations with the greatest healthcare needs. Utilizing the model, Health Districts can transition from a reactive logic to a proactive stratification of demand. Rather than relying solely on demographic data, Health Districts should adopt a vulnerability-weighted approach to guide targeted health interventions in high-need areas. This strategy would support the deployment territorial networks based on predicted service demand, ensuring that universal coverage translates into equitable access.

This study has several limitations that should be acknowledged. First, its ecological design implies that all analyses were conducted at an aggregated territorial level rather than at the individual level. While this approach is suitable for population health planning, it introduces the risk of ecological fallacy, whereby associations observed at the group level may not accurately reflect individual-level relationships. Consequently, causal inferences cannot be drawn.

Second, the study relies on cross-sectional data, which limits the ability to establish temporal or causal relationships between socioeconomic determinants and mortality outcomes. The observed associations should therefore be interpreted as descriptive rather than causal.

Furthermore, as socioeconomic indicators are derived from the 2021 Census, whereas mortality data refer to the 2023–2024 period, a potential limitation is that post-pandemic socioeconomic changes may not be fully captured by the 2021 data. Nevertheless, the census remains the most recent and authoritative population-level source available.

Third, although the model integrates multiple socioeconomic indicators, the clinical dimension is only indirectly represented through mortality (SMR) and lacks detailed patient-level information such as comorbidities, disease severity, healthcare utilization, or behavioral risk factors. This limited clinical stratification may lead to residual confounding and does not fully capture the complexity of health needs within populations.

Although the study demonstrates solid predictive power, its findings should be interpreted with caution. Aggregating municipal data into 1175 units helped stabilize the SMR distribution, but it may also obscure intra-unit heterogeneity, particularly in metropolitan areas where affluent and deprived neighborhoods coexist. Indeed, the aggregation of municipalities into larger territorial units, while necessary to reduce stochastic variability, might result in a loss of local heterogeneity, potentially masking intra-area disparities, especially in highly diverse or urban settings.

In our study we found a residual variance of 27.5% that represents the proportion of variability not explained by the variables included in the model, suggesting that additional unmeasured factors—potentially environmental—could also contribute to the observed outcomes. In this context, several Italian studies have sought to clarify the biological mechanisms underlying the observed associations between air pollution exposure and adverse health outcomes. This body of research suggests that environmental stressors, such as poor air quality and industrial emissions, contribute to less healthy living conditions, thereby exposing populations to higher levels of harmful environmental exposures [24,34,35].

Additionally, the decentralized structure of the NHS can complicate comparisons across the 21 regional health systems, as socioeconomic drivers may interact with differing administrative efficiencies or varying levels of private-sector involvement. The asynchrony between current mortality data (2023–2024) and structural indicators means that the most recent economic shifts may not be fully captured.

Finally, the use of administrative and secondary data sources may be subject to data quality issues, including measurement errors, reporting biases and variability in data completeness across regions.

Despite these limitations, the study provides a framework for territorial health analysis and offers valuable insights for population health management and healthcare planning, and the model demonstrates considerable strengths. It offers a robust analytical and replicable foundation for advancing the proximity-based objectives outlined in DM 77/2022, supporting the sustainability of the Italian NHS by facilitating a proactive approach to addressing the social determinants that shape healthcare demand.

## 5. Conclusions

Our model confirms that mortality in Italy is not merely the result of biological factors, but is profoundly shaped by the territorial context. By transitioning from a reactive, hospital-centric model to a proactive, equity-based stratification, the Italian NHS can finally operationalize the principles of the PNRR and DM 77/2022 [22,36].

Since the Health District is intended to be the primary unit responsible for service planning, based on rigorous population needs assessments, leveraging the model score as a structural rebalancing tool ensures that service availability aligns with territorial vulnerability rather than simple population density. Crucially, this approach empowers regional programming, providing a data-driven baseline to ensure that regional autonomy does not translate into geographical inequity. This analytical foundation is essential for building a sustainable, proximity-based NHS that ensures that universal coverage translates into truly equitable access.

## Figures and Tables

**Table 1:** Descriptive statistics of population size (P1) and socioeconomic deprivation indicators (L1–L4b) *.

Territorial Units (*n* = 1175)	Population (P1)	Education (L1%)	Employment (L2%)	Citizenship (L3%)	Household Density (L4)	Isolation (L4a %)	Overcrowding (L4b %)
Mean	50,188.11	51.09	65.47	6.95	2.23	37.11	1.14
Standard error	3293.39	0.19	0.35	0.12	0.006	0.21	0.02
Median	29,234	51.16	68.65	6.50	2.23	36.01	1.04
SD	112,891.641	6.54	12.09	4.08	0.205	7.17	0.57
Sample Variance	12,744,522,559.77	0.43	1.46	0.17	0.042	0.51	0.00
Kurtosis	301,671	0.123	−1.116	1.3	1.093	1.76	2.68
Skewness	14,445	0.193	0.204	0.855	−0.179	0.870	1.224
Minimum	177	33.73	34.78	0.53	1.48	18.9	0.00
Maximum	2,751,747	76.56	100	28.12	2.87	67.8	4.71

* L1 (education): % of population ≥9 years with education below upper secondary school; L2 (Employment): % of the active population who is occupied, L3 (Citizenship): % of foreign residents, L4 (Household density), L4a (Isolation): % of housing units with a single occupant; L4b (Overcrowding): % of housing units with 5+ occupants.

**Table 2:** Population-weighted multivariable linear regression with fixed effects for region and SNAI area (2023–2024).

	Main Analysis				Sensitivity Analysis			
Variable/Parameter	β (*p*-Value)		IC 95%		β (*p*-Value)		IC 95%	
Intercept	0.87	<0.0001	0.77	0.96	0.89	<0.0001	0.80	0.98
Education (L1)	0.59	<0.0001	0.49	0.68	0.55	<0.0001	0.47	0.64
Employment (L2)	−0.38	<0.0001	−0.47	−0.29	−0.36	<0.0001	−0.44	−0.27
Isolation (L4a *)	0.29	<0.0001	0.20	0.38	0.24	<0.0001	0.16	0.31
SNAI [A]	0.01	0.1838	0.00	0.01	--	--	--	--
SNAI [B]	−0.01	0.5391	−0.02	0.01	--	--	--	--
SNAI [C]	0.01	0.0762	0.00	0.01	--	--	--	--
SNAI [D]	0.01	0.0153	0.00	0.02	--	--	--	--
Regional fixed effects	Yes				Yes			
SNAI Area classification fixed effects	Yes				No			
Global test for SNAI area classification fixed effects	F = 4.3267; *p* = 0.0018				—			
Global test for Region	*p* < 0.0001				*p* < 0.0001			
Number of territorial units	1.175				1.175			
Weighted population	58,971,031				58,971,031			
R^2^	0.725				0.721			
Adjusted R^2^	0.719				0.716			
Overall model F-test	F = 116.5423; *p* < 0.0001				F = 135.3813; *p* < 0.0001			

Notes: All models were fitted using ordinary least squares (OLS) regression with analytic weights proportional to the resident population of each territorial unit. Beta coefficients represent the change in SMR associated with a one unit increase (1 percentage point) in each deprivation indicator. Main Analysis: Includes deprivation indicators (L1, L2, L4a), regional fixed effects, and SNAI area classification fixed effects. * Sensitivity Analysis: Excludes SNAI area classification to test the stability of the primary deprivation estimates. Model Diagnostics: The inclusion of SNAI area resulted in a statistically significant contribution to the model (Joint F-test = 4.33, *p* = 0.0018) and a modest increase in the explained variance (R^2^ from 0.721 to 0.725). Reference Categories: Veneto for Regional fixed effects and Class E-F for SNAI area classification. Abbreviations: CI, confidence interval; OLS, ordinary least squares; SNAI, Strategia Nazionale Aree Interne; SMR, Standardized Mortality Ratio.

## Data Availability

Data can extracted from ISTAT. Indicators of Socio-Economic Disadvantage and Territorial Vulnerability. 2021. https://www.istat.it/comunicato-stampa/dati-disagio-socio-economico-livello-sub-comunale-idise-anno-2021 (accessed on 10 February 2026).

## Supplementary Materials

The following supporting information can be downloaded at: https://www.mdpi.com/article/10.3390/healthcare14111456/s1; Table S1: STROBE (Strengthening the Reporting of Observational Studies in epidemiology) checklist for cross-sectional studies; Table S2: Study analytical workflow; Table S3: Descriptive statistics of socioeconomic indicators and population distribution; Table S4: Population-weighted correlation matrix of socioeconomic indicators (L1–L4b) and Standardized Mortality Ratio (SMR) 2023–2024—Cluster DA; Table S5 Full Parameter Estimates: Main Analysis Model (including Area PAI/SNAI); Table S6: Summary of Fit: Main Analysis Model; Table S7: Analysis of Variance (ANOVA): Main Analysis Model; Table S8: Full Parameter Estimates: Sensitivity Analysis Model (excluding SNAI); Table S9: Summary of Fit and Model Diagnostics (Sensitivity Model); Table S10: Analysis of Variance (ANOVA).
