# Supplementary material for: Bridging the Gap Between Social Determinants and Health Profile: A New Stratification Tool for the Italian National Health Service
Source: Healthcare (Basel). 2026 May 25;14(11):1456. doi: 10.3390/healthcare14111456 (PMC13256984; doi:10.3390/healthcare14111456)
Supplement: Supplementary file 1 [file healthcare-14-01456-s001.zip › healthcare-4190579-supplementary.pdf]

Table S1. STROBE (strengthening the reporting of observational studies in epidemiology) checklist for cross-sectional studies

Item	No	Recommendation	Reported in section/subsection (§)
Title and abstract	1	(a) Indicate the study's design with a commonly used term in the title or the abstract	Title; Abstract
		(b) Provide in the abstract an informative and balanced summary of what was done and what was found	Abstract
Introduction			
Background/rationale	2	Explain the scientific background and rationale for the investigation being reported	Introduction (1–3)
Objectives	3	State specific objectives, including any prespecified hypotheses	Introduction (3)
Methods			
Study design	4	Present key elements of study design early in the paper	Methods/Study design and setting (1)
Setting	5	Describe the setting, locations, and relevant dates, including periods of recruitment, exposure, follow-up, and data collection	Methods/Study design and setting (2, 3); Methods/Data source and eligibility criteria (1)
Participants	6	(a) Give the eligibility criteria, and the sources and methods of selection of participants	Methods/Data source and eligibility criteria (1, 2)
Variables	7	Clearly define all outcomes, exposures, predictors, potential confounders, and effect modifiers. Give diagnostic criteria, if applicable	Methods/Study outcome (1); Methods/Study variables (1, 2)
Data sources/ measurement	8	For each variable of interest, give sources of data and details of methods of assessment (measurement). Describe comparability of assessment methods if there is more than one group	Study design and setting (3); Methods/Study outcome (1); Methods/Study variables (1, 2)
Bias	9	Describe any efforts to address potential sources of bias	Methods/Data source and eligibility criteria (2)
Study size	10	Explain how the study size was arrived at	Methods/Study outcome (1); Methods/Data source and eligibility criteria (2)
Quantitative variables	11	Explain how quantitative variables were handled in the analyses. If applicable, describe which groupings were chosen and why	Methods/Statistical analysis (1)
Statistical methods	12	(a) Describe all statistical methods, including those used to control for confounding	Methods/Statistical analysis (1)
		(b) Describe any methods used to examine subgroups and interactions	Methods/Statistical analysis (1)
		(c) Explain how missing data were addressed	Methods/Study variables (2)
		(d) If applicable, describe analytical methods taking account of sampling strategy	Not applicable
		(e) Describe any sensitivity analyses	Methods/Study outcome (1)
Results			
Participants	13	(a) Report numbers of individuals at each stage of study—e.g. numbers potentially	Results/Description of the study population (1); Results/Effects of

		eligible, examined for eligibility, confirmed eligible, included in the study, completing follow-up, and analyzed	citizenship on non-urgent emergency department accesses (1)
		(b) Give reasons for non-participation at each stage	Results/Effects of citizenship on non-urgent emergency department accesses (1)
		(c) Consider use of a flow diagram	Fig. 1
Descriptive data	14*	(a) Give characteristics of study participants (e.g. demographic, clinical, social) and information on exposures and potential confounders	Results/Description of the study population (1, 2); Table 1
		(b) Indicate number of participants with missing data for each variable of interest	Results/Effects of citizenship on non-urgent emergency department accesses (1)
Outcome data	15*	Report numbers of outcome events or summary measures	Results/Effects of citizenship on non-urgent emergency department accesses (2); Fig. 1; Fig. 2
Main results	16	(a) Give unadjusted estimates and, if applicable, confounder-adjusted estimates and their precision (e.g., 95% confidence interval). Make clear which confounders were adjusted for and why they were included	Results/Effects of citizenship on non-urgent emergency department accesses (2, 5); Table 2; Table S2; Table S3
		(b) Report category boundaries when continuous variables were categorized	Not applicable
		(c) If relevant, consider translating estimates of relative risk into absolute risk for a meaningful time period	Not applicable
Other analyses	17	Report other analyses done—e.g. analyses of subgroups and interactions, and sensitivity analyses	Results/Effects of citizenship on non-urgent emergency department accesses (3–5); Table 2; Fig. 2; Fig. 3; Fig. 4; Table S4; Table S5
Discussion			
Key results	18	Summarize key results with reference to study objectives	Discussion (1)
Limitations	19	Discuss limitations of the study, taking into account sources of potential bias or imprecision. Discuss both direction and magnitude of any potential bias	Discussion (5)
Interpretation	20	Give a cautious overall interpretation of results considering objectives, limitations, multiplicity of analyses, results from similar studies, and other relevant evidence	Discussion (5)
Generalizability	21	Discuss the generalizability (external validity) of the study results	Discussion (2–5); Conclusions (1)
Other information			
Funding	22	Give the source of funding and the role of the funders for the present study and, if applicable, for the original study on which the present article is based	Funding

Table S2. Study analytical workflow

DATA INPUT:

Municipalities and population [Italian National Institute of Statistics (ISTAT), 2024]

Socioeconomic data [Italian National Institute of Statistics (ISTAT), Permanent Census of Population and Housing Data, 2021]

Health District boundaries [Law No. 833/1978; Legislative Decrees No. 502/1992, No. 229/1999, Gabrielli et al. 2024]

National Strategy for Inner Areas (SNAI) classification [Italian Presidency of the Council of Ministers. National Strategic Plan for Inland Areas (PSNAI) 2021–2027]

Mortality data [Italian National Institute of Statistics ISTAT, 2023-2024]

↓

DATA TRANSFORMATION:

Standardized mortality data

[indirect standardization for population (std: Italy)]

Socioeconomic profile: definition of Key indicators

[Caranci et al. 2010, Rosano et al. 2020]

L1 (Education): % of population ≥9 years with education below upper secondary school;

L2 (Employment): % of the active population who is occupied;

L3 (Citizenship): % of foreign residents;

L4 (Household density): average number of occupants per housing unit;

L4a (Isolation): % of housing units with a single occupant;

L4b (Overcrowding): % of housing units with 5+ occupants.

↓

DATA CLEANING AND AGGREGATION:

Verification of missing data, formal check of percentages, aggregation per District and PAI

↓

DATA ANALYSIS:

Exploratory: Correlation matrices, Descriptive stats. Inference: Population-weighted OLS regression.

Table S3. Descriptive statistics of socioeconomic indicators and population distribution.

Municipalities (N=7,899)	Population (P1)	Education (L1 %)	Employment (L2 %)	Social isolation (L4a %)
Mean	7,466	52.43	66.85	37.48
Standard Error	468	0.08	0.13	0.10
Median	2,392	52.21	70.07	35.74
SD	41,608	6.77	11.27	8.75
Kurtosis	2,605.02	0.50	-0.74	2.03
Minimum	33	23.68	31.38	16.59
Maximum	2,751,747	83.92	100.00	90.91

Table S4. Population-weighted correlation matrix of socioeconomic indicators (L1–L4b) and Standardized Mortality Ratio (SMR) 2023–2024 – Cluster DA

	**L1**	**L2**	**L3**	**L4**	**L4a**	**L4b**	**SMR 2023**	**SMR 2024**	**SMR 2023-2024**
L1	1.0000	-0.5582	-0.5378	0.6085	-0.6391	0.3489	0.4383	0.4892	0.4841
L2	-0.5582	1.0000	0.8400	-0.5367	0.4515	-0.1576	-0.7333	-0.6920	-0.7465
L3	-0.5378	0.8400	1.0000	-0.5258	0.5403	-0.0175	-0.4876	-0.4869	-0.5098
L4	0.6085	-0.5367	-0.5258	1.0000	-0.9576	0.7294	0.3952	0.4294	0.4307
L4a	-0.6391	0.4515	0.5403	-0.9576	1.0000	-0.6137	-0.2923	-0.3402	-0.3299
L4b	0.3489	-0.1576	-0.0175	0.7294	-0.6137	1.0000	0.2400	0.2876	0.2750
SMR 2023	0.4383	-0.7333	-0.4876	0.3952	-0.2923	0.2400	1.0000	0.8268	0.9593
SMR 2024	0.4892	-0.6920	-0.4869	0.4294	-0.3402	0.2876	0.8268	1.0000	0.9520
SMR 2023-2024	0.4841	-0.7465	-0.5098	0.4307	-0.3299	0.2750	0.9593	0.9520	1.0000

Table S5. Full Parameter Estimates: Main Analysis Model (including Area PAI/SNAI)

**Variable/parameter**	**Estimates**	**Std Error**	**T-Ratio**	**Prob>|t|**	**CI inf 95%**	**CI sup 95%**
Intercept	0.8668938	0.048622	17.83	<.0001	0.7714967	0.962291
L1	0.5875385	0.048908	12.01	<.0001	0.4915789	0.6834982
L2	-0.381032	0.045457	-8.38	<.0001	-0.470221	-0.291843
L4a	0.2909145	0.045041	6.46	<.0001	0.2025424	0.3792866
Region						
Abruzzo	0.0252381	0.011466	2.20	0.0279	0.002741	0.0477352
Basilicata	-0.003686	0.017651	-0.21	0.8346	-0.038318	0.0309448
Calabria	0.0252423	0.01177	2.14	0.0322	0.0021497	0.048335
Campania	0.1228159	0.009284	13.23	<.0001	0.1045997	0.1410322
Emilia-Romagna	0.013352	0.008419	1.59	0.1130	-0.003167	0.0298713
Friuli-Venezia Giulia	-0.009966	0.012089	-0.82	0.4099	-0.033685	0.0137518
Lazio	0.0513439	0.006394	8.03	<.0001	0.0387984	0.0638893
Liguria	-0.000703	0.010878	-0.06	0.9485	-0.022046	0.0206397
Lombardy	-0.014108	0.007061	-2.00	0.0460	-0.027963	-0.000253
Marche	-0.031694	0.010807	-2.93	0.0034	-0.052897	-0.010491
Molise	0.0564522	0.023254	2.43	0.0154	0.0108265	0.1020779
Piedmont	0.0110208	0.007698	1.43	0.1525	-0.004083	0.0261246
Apulia	-0.054298	0.00883	-6.15	<.0001	-0.071622	-0.036973
Sardinia	-0.09001	0.011507	-7.82	<.0001	-0.112586	-0.067434
Sicily	0.0320207	0.010008	3.20	0.0014	0.0123853	0.0516562
Tuscany	-0.021485	0.008271	-2.60	0.0095	-0.037713	-0.005257
Trentino-Alto Adige/Südtirol	-0.033212	0.012202	-2.72	0.0066	-0.057154	-0.009271

Umbria	-0.000553	0.013825	-0.04	0.9681	-0.027678	0.0265728
Valle d'Aosta/Vallée d'Aoste	-0.034867	0.035144	-0.99	0.3213	-0.103821	0.0340861
SNAI (A)	0.005834	0.004386	1.33	0.1838	-0.002772	0.0144399
SNAI (B)	-0.005217	0.008492	-0.61	0.5391	-0.021879	0.0114455
SNAI (C)	0.0069296	0.003904	1.77	0.0762	-0.00073	0.0145895
SNAI (D)	0.010837	0.004461	2.43	0.0153	0.0020835	0.0195906

Notes: - Veneto is the reference category for Region; Class E-F is the reference for Area PAI (SNAI).

The table reports the complete set of regression coefficients for the primary multivariable model. By including Area PAI as a fixed effect, this model accounts for the specific territorial classification (SNAI) in addition to regional administrative boundaries. This provides further adjustment for geographical heterogeneity in the association between socioeconomic indicators and mortality.The Area PAI/SNAI fixed effects were jointly significant in the primary model (joint F-test: F = 4.33, p = 0.0018), suggesting that territorial classification contributed additional explanatory information beyond socioeconomic indicators and regional fixed effects.

Table S6. Summary of Fit: Main Analysis Model

Source	Nparm	DF	Sum of Squares	F Ratio	Prob > F
L1-Educational Deprivation	1	1	23763.89	144.3137	<.0001
L2- Employment-related deprivation	1	1	11569.76	70.2610	<.0001
L4a- Social isolation	1	1	6869.46	41.7170	<.0001
Region	19	19	104912.75	33.5324	<.0001
SNAI	4	4	2849.87	4.3267	0.0018

Goodness-of-fit statistics for the primary model. The comparison between R-square and Adjusted R-square confirms that the model is parsimonious despite the high number of geographical fixed effects.

Table S7. Analysis of Variance (ANOVA): Main Analysis Model

Source	DF	Sum of Squares	Mean Square	F Ratio
Model	26	498960.96	19190.8	116.5423
Error	1148	189039.12	164.7	Prob > F
C. total	1174	688000.08		<.0001

Systematic breakdown of the variance. The F Ratio tests the overall significance of the model including deprivation indices. regional effects. and Area PAI categories.

Table S8. Full Parameter Estimates: Sensitivity Analysis Model (excluding SNAI)

Variable/parameter	Estimates	Std Error	T-Ratio	Prob>|t|	CI inf 95%	CI sup 95%
Intercept	0.8894392	0.047273	18.81	<.0001	0.7966884	0.9821901
L1-Educational Deprivation	0.5520696	0.043345	12.74	<.0001	0.4670257	0.6371135
L2- Employment-related deprivation	-0.355411	0.045019	-7.89	<.0001	-0.44374	-0.267081
L4a- Social isolation	0.2387441	0.037967	6.29	<.0001	0.1642517	0.3132364
Region						
Abruzzo	0.0242212	0.011497	2.11	0.0354	0.0016639	0.0467786
Basilicata	-0.011226	0.017564	-0.64	0.5228	-0.045686	0.0232341
Calabria	0.0285665	0.011764	2.43	0.0153	0.0054856	0.0516475
Campania	0.1253496	0.009074	13.81	<.0001	0.1075463	0.143153
Emilia-Romagna	0.0121873	0.008451	1.44	0.1496	-0.004395	0.0287691
Friuli-Venezia Giulia	-0.009864	0.012079	-0.82	0.4143	-0.033563	0.0138358
Lazio	0.0526709	0.006399	8.23	<.0001	0.0401168	0.0652251
Liguria	0.0035836	0.010777	0.33	0.7396	-0.017561	0.0247284
Lombardy	-0.015632	0.006993	-2.24	0.0256	-0.029353	-0.001911
Marche	-0.033474	0.010858	-3.08	0.0021	-0.054778	-0.01217
Molise	0.0497037	0.023293	2.13	0.0331	0.0040027	0.0954047
Piedmont	0.0126503	0.007618	1.66	0.0971	-0.002296	0.0275972
Apulia	-0.051001	0.008597	-5.93	<.0001	-0.067868	-0.034135
Sardinia	-0.085068	0.011353	-7.49	<.0001	-0.107343	-0.062793
Sicily	0.0352582	0.009704	3.63	0.0003	0.0162188	0.0542976
Tuscany	-0.023062	0.008297	-2.78	0.0055	-0.03934	-0.006783
Trentino-Alto Adige/Südtirol	-0.037628	0.011635	-3.23	0.0013	-0.060456	-0.014801
Umbria	-0.002795	0.01387	-0.20	0.8403	-0.030008	0.0244181
Valle d'Aosta/Vallée d'Aoste	-0.030014	0.03528	-0.85	0.3951	-0.099233	0.0392057

Notes: - Veneto is the reference category for Region variables

This sensitivity model excludes Area PAI/SNAI fixed effects and reports the full set of regression coefficients (β). standard errors. 95% confidence intervals. and individual p-values. The model was used to evaluate whether the associations between socioeconomic indicators and SMR remained stable after omitting adjustment for territorial accessibility classification.

Table S9. Summary of Fit and Model Diagnostics (Sensitivity Model)

Source	Nparm	DF	Sum of Squares	F Ratio	Prob > F
L1-Educational Deprivation	1	1	27021.42	162.2223	<.0001
L2- Employment-related deprivation	1	1	10381.48	62.3249	<.0001
L4a- Social isolation	1	1	6586.41	39.5413	<.0001
Region	19	19	105781.79	33.4241	<.0001

The table reports the goodness-of-fit metrics for the sensitivity analysis. The Adjusted R-squared accounts for the number of predictors (Regional Fixed Effects). providing a conservative estimate of the variance explained by the model.

Table S10. Analysis of Variance (ANOVA)

Source	DF	Sum of Squares	Mean Square	F Ratio
Model	22	496111.09	22550.5	135.3813
Error	1152	191888.99	166.6	**Prob > F**
C. total	1174	688000.08		<.0001

The ANOVA table partitions the total sum of squares into the component explained by the regression model and the residual (error) component. The F Ratio tests the null hypothesis that all regression coefficients (excluding the intercept) are equal to zero.
